# Vitamin D Receptor Deficiency Upregulates Pulmonary Artery Kv7 Channel Activity

**DOI:** 10.3390/ijms241512350

**Published:** 2023-08-02

**Authors:** Miguel A. Olivencia, Marta Villegas-Esguevillas, Maria Sancho, Bianca Barreira, Elena Paternoster, Rui Adão, María Jesús Larriba, Angel Cogolludo, Francisco Perez-Vizcaino

**Affiliations:** 1 Department of Pharmacology and Toxicology, School of Medicine, University Complutense of Madrid, 28040 Madrid, Spain; mioliven@ucm.es (M.A.O.); martvi05@ucm.es (M.V.-E.); biancabarreira@med.ucm.es (B.B.); elepater@ucm.es (E.P.); ruimigueladao@gmail.com (R.A.); acogolludo@med.ucm.es (A.C.); 2 Ciber Enfermedades Respiratorias (CIBERES), 28029 Madrid, Spain; masanc75@ucm.es; 3 Instituto de Investigación Sanitaria Gregorio Marañón (IiSGM), 28009 Madrid, Spain; 4 Department of Physiology, School of Medicine, Universidad Complutense de Madrid, 28040 Madrid, Spain; 5 Instituto de Investigaciones Biomédicas Alberto Sols, Consejo Superior de Investigaciones Científicas, Universidad Autónoma de Madrid, 28029 Madrid, Spain; 6 Ciber Cáncer (CIBERONC), 28029 Madrid, Spain; 7 Instituto de Investigación Sanitaria del Hospital Universitario La Paz (IdiPAZ), 28029 Madrid, Spain

**Keywords:** vitamin D receptor, pulmonary hypertension, Kv7 channels, KCNE

## Abstract

Recent evidence suggests that vitamin D is involved in the development of pulmonary arterial hypertension (PAH). The aim of this study was to analyze the electrophysiological and contractile properties of pulmonary arteries (PAs) in vitamin D receptor knockout mice (*Vdr^−^*^/*−*^). PAs were dissected and mounted in a wire myograph. Potassium membrane currents were recorded in freshly isolated PA smooth muscle cells (PASMCs) using the conventional whole-cell configuration of the patch-clamp technique. Potential vitamin D response elements (VDREs) in Kv7 channels coding genes were studied, and their protein expression was analyzed. *Vdr^−^*^/*−*^ mice did not show a pulmonary hypertensive phenotype, as neither right ventricular hypertrophy nor endothelial dysfunction was apparent. However, resistance PA from these mice exhibited increased response to retigabine, a Kv7 activator, compared to controls and heterozygous mice. Furthermore, the current sensitive to XE991, a Kv7 inhibitor, was also higher in PASMCs from knockout mice. A possible VDRE was found in the gene coding for KCNE4, the regulatory subunit of Kv7.4. Accordingly, *Vdr^−^*^/*−*^ mice showed an increased expression of KCNE4 in the lungs, with no changes in Kv7.1 and Kv7.4. These results indicate that the absence of *Vdr* in mice, as occurred with vitamin D deficient rats, is not sufficient to induce PAH. However, the contribution of Kv7 channel currents to the regulation of PA tone is increased in Vdr^−/−^ mice, resembling animals and humans suffering from PAH.

## 1. Introduction

Pulmonary arterial hypertension (PAH) is a severe condition characterized by abnormally high mean pulmonary arterial pressure (mPAP) due to persistent vasoconstriction, proliferation, and inflammation of the pulmonary arteries (PAs) [1,2]. Potassium channels of the voltage-dependent (Kv) or K2P superfamilies play an important role in pulmonary circulation by regulating membrane potential and arterial tone, as well as cell proliferation and the survival of pulmonary artery smooth muscle cells (PASMCs). Ionic channel remodeling, which is mainly characterized by dysfunctional Kv1.5 and TASK-1 channels, is a common feature of PAH in animal models and humans [3,4,5,6,7,8].

Kv7 channels are also known as key regulators of vascular tone in health and disease [9,10,11]. Activation of Kv7 causes a K^+^ outward efflux and, thereby, hyperpolarization and relaxation, as well as antiproliferative effects in vascular smooth muscle cells. Different vasodilator and vasoconstrictor agents, including endogenous and pharmacological activators of the cAMP and cGMP pathways, regulate Kv7 channel activity [9,10,12]. The pore-forming α-subunits of Kv channels, Kv7.1, Kv7.4, and Kv7.5, as well as the modulatory β subunit KCNE4, are expressed in vascular smooth muscle cells. KCNE4 modulates K^+^ channel function by increasing Kv7.4 activity and membrane expression [13,14,15,16]. In fact, Mondejar-Parreño et al. demonstrated that increased KCNE4 expression resulted in higher Kv7 contribution to overall K^+^ conductance and vascular tone in pulmonary arteries in PAH [14].

1,25-dihydroxyvitamin D, the most active vitamin D metabolite, is a liposoluble hormone that, in addition to the regulation of calcium–phosphorus homeostasis, exerts control over cell growth, differentiation and migration, intracellular metabolism, vascular tone, and immunity, among other functions [17,18]. It binds to the nuclear vitamin D receptor (VDR, encoded by the *VDR* and *Vdr* genes in humans and mice, respectively) to regulate the expression of specific target genes [19]. Vitamin D deficiency is now recognized as a pandemic [19] and has been related to increased all-cause and cardiovascular mortality in epidemiological studies [20]. Furthermore, compared to the general population or individuals with other cardiovascular disorders, vitamin D deficiency is more frequent in PAH patients [21,22]. Callejo et al. demonstrated that vitamin D deficiency is associated with poor prognosis in PAH patients and with aggravation of disease in a rat model of PAH [21,23]. In addition, ionic remodeling has been shown in vitamin-D-deficient rats with reduced TASK-1 lung expression and decreased TASK-like potassium currents [23].

However, the role of VDR in the pulmonary vasculature is not well characterized. A possible role for VDR in maintaining pulmonary barrier integrity has been described because *Vdr^−^*^/*−*^ mice present alterations in adherent junctions in the lungs [24]. In addition, previous studies have demonstrated the presence and functionality of VDR in bronchial epithelial cells and alveolar macrophages [24,25,26]. Interestingly, low lung VDR expression has been found in patients with acute lung damage, chronic obstructive pulmonary disease, and idiopathic pulmonary fibrosis. Furthermore, decreased VDR levels have been correlated with a worse prognosis [27,28]. We also found lower expression of VDR in lungs from PAH patients [29], although the significance of this result is unknown.

The aim of the present study is to explore the role of *Vdr* in the pulmonary vasculature of mice. Therefore, we analyzed whether *Vdr* ablation in mice induces pulmonary arterial dysfunction.

## 2. Results

### 2.1. Effects of Vdr Ablation on RV Weight and Endothelial Function

**Figure 1:**
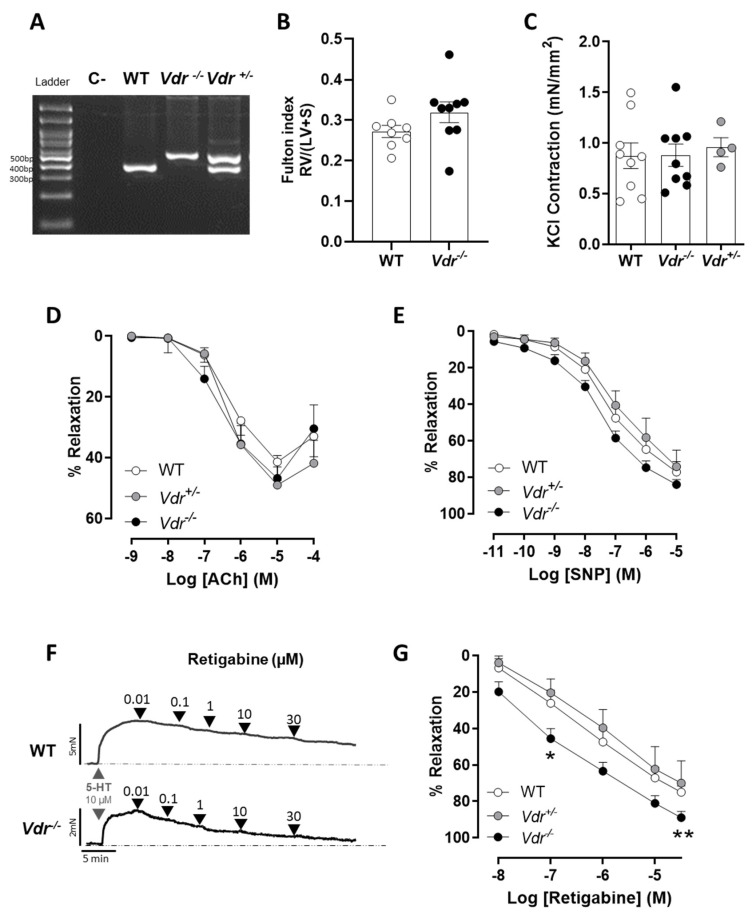
Right ventricular hypertrophy and pulmonary vascular function in *Vdr^−^*^/*−*^ mice. (**A**) Representative genotyping of WT, *Vdr^−^*^/*−*^, and *Vdr^+^*^/*−*^ mice. *Vdr^−^*^/*−*^ animals have an insert in the *Vdr* gene that increases the base pairs, resulting in amplification of the gene. (**B**) Fulton index (RV/(LV+S)). (**C**) Maximum PA contraction to KCl. (**D**–**G**) Relaxant responses in WT, *Vdr^+^*^/*−*^, and *Vdr^−^*^/*−*^ PAs to (**D**) acetylcholine, (**E**) sodium nitroprusside, and (**F**,**G**) retigabine. Panel F shows an original representative trace of the retigabine dose–response curve. Results are means ± standard error of the mean (n = 9 WT, 9 *Vdr^−^*^/*−*^ and 4 *Vdr^+^*^/*−*^). * *p* < 0.05, ** *p* < 0.01 vs. WT using a two-way ANOVA test followed by a Bonferroni post hoc test.

Heterozygous *Vdr*^+/−^ animals are phenotypically normal in terms of calcium and phosphorus homeostasis, as well as bone development and hair growth [30]. *Vdr*^−/−^ mice do not express the VDR protein [31] and were found to be viable when supplemented with calcium, phosphorus, and lactose, although they were alopecic and had lower body weight and a reduced survival rate after 6 months [31,32]. We failed to keep them stable after anesthesia, mechanical ventilation, and open-chest surgery to achieve a reliable hemodynamic recording. Therefore, we did not expose them to hypoxia plus Su5416 to induce PAH as we initially aimed. Consequently, we analyzed the mice for possible right ventricular hypertrophy, a common consequence of PAH in both animals and humans, as a surrogate marker of elevated mPAP. *Vdr* knockout mice did not show increased RV weight expressed as the left ventricular + septum (LV+S) weight, i.e., the Fulton index, when compared to wild-type mice (Figure 1B). Heterozygous *Vdr*^+/−^ animals were phenotypically normal. Additionally, there were no changes in maximum PA contraction to 80 mM KCl (Figure 1C), endothelial-dependent relaxation to acetylcholine (Figure 1D), or endothelial-independent relaxation to SNP in *Vdr*^−/−^ mice (Figure 1E).

### 2.2. Kv7 Activity in Vdr^−/−^ Pulmonary Vasculature

**Figure 2:**
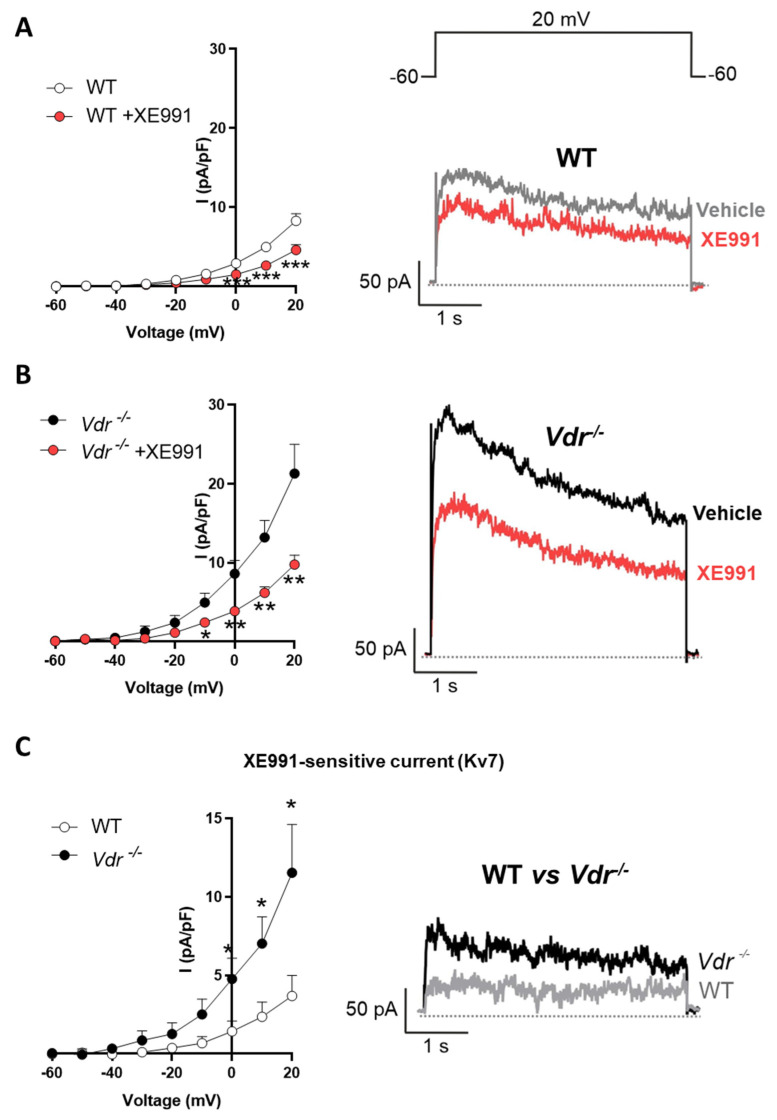
Kv7 current in *Vdr^−^*^/*−*^ pulmonary artery smooth muscle cells (PASMCs). (**A**,**B**) K^+^ current–voltage relationships measured before and after the addition of XE991 (10 µmol/L). Insets show representative current traces measured at +20 mV in the absence and presence of XE991 in (**A**) WT and (**B**) *Vdr^−^*^/*−*^ PASMCs. (**C**) XE991-sensitive current obtained by subtracting the K^+^ current in the presence of XE991 from the current in the absence of the drug. Insets show representative XE991-sensitive current traces measured at +20 mV. Results are means ± standard error of the mean (n = 4 animals and 8 cells per group). * *p* < 0.05, ** *p* < 0.01, *** *p* < 0.001 vs. WT using a two-way ANOVA test followed by a Bonferroni post hoc test.

The Kv7 activator retigabine induces a concentration-dependent vasodilation in PAs (Figure 1F). Interestingly, PAs from *Vdr* knockout mice exhibited significantly higher relaxation in response to retigabine when compared to WT mice (Figure 1G). Thus, Kv currents were recorded in fresh PASMCs to study the role of *Vdr* in the regulation of voltage-gated potassium channels. The specific Kv7 inhibitor XE991 was used to characterize the contribution of the Kv7 channels to the total K^+^ current. In PASMCs isolated from both control (Figure 2A) and *Vdr*^−/−^ mice (Figure 2B), K^+^ currents were decreased in the presence of XE991. However, the amplitude of the XE991-sensitive current, as calculated by subtracting the current in the absence and in the presence of XE991, was significantly higher in PASMCs from *Vdr*^−/−^ mice, as shown in Figure 2C.

### 2.3. Lung Histology

**Figure 3:**
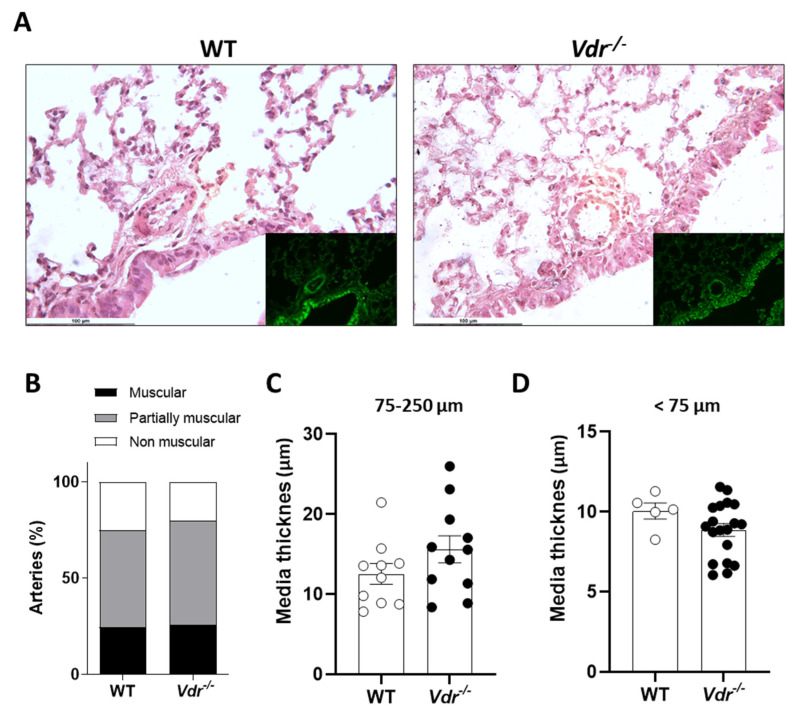
Lung histology. (**A**) Representative lung section images stained with hematoxylin and eosin and examined by light microscopy; the insets show elastin visualized by its green autofluorescence. (**B**) Percentage of muscular (black), partially muscular (grey), and non-muscular (white) vessels (n = 4 mice per group, 1–5 vessels analyzed per mouse). (**C**,**D**) PA medial wall thickness of 75–250 µm and <75 µm, respectively.

Pulmonary vasculature morphology and remodeling were analyzed in hematoxylin/eosin-stained lung sections (Figure 3). Small PAs were classified in a blinded fashion as muscular, partially muscular, and non-muscular arteries. We found no changes in the percentage of muscularized arteries or in the medial wall thickness.

### 2.4. VDRE in the KCNE4 Gene

**Figure 4:**
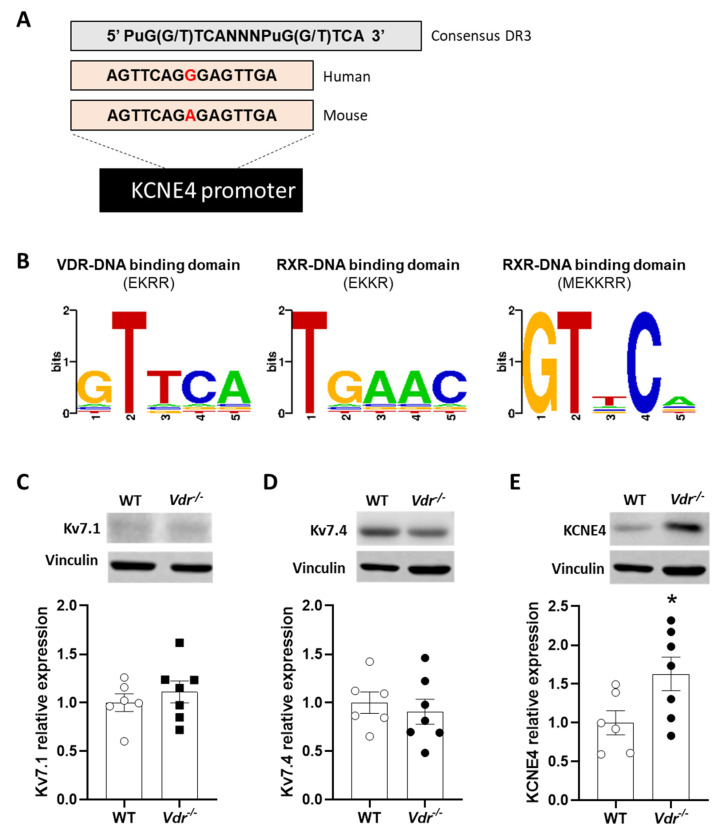
*KCNE4* vitamin D response element (VDRE) and Kv7 channel expression in *Vdr* knockout mice. (**A**) Predicted VDRE in the promoter region of the *KCNE4* gene conserved between humans and mice. (**B**) Vitamin D receptor (VDR) and retinoid X receptor (RXR) protein interface signatures and the estimated binding specificities in the VDRE of the *KCNE4* gene as sequence logo provided by 3D-footprint. Larger letters indicate greater affinity, and smaller letters indicate the allowable variability. (**C**–**E**) Averaged densitometric protein expression of (**C**) Kv7.1, (**D**) Kv7.4, and (**E**) KCNE4. Results are means ± standard error of the mean (n = 6–7 animals). * *p* < 0.05 vs. WT using a *t*-test. Original Western Blotting data can be found in Figure S1.

We searched the database for potential DNA motifs contained within the *KCNE4* gene promoter comprising the consensus VDRE sequence formed by direct or everted repeats of PuG(G/T)TCA separated by 3 or 6 bp (DR3 or ER6), including those that varied from the consensus sequence by a single nucleotide substitution [23]. Four DR3 elements were found in the human *KCNE4* gene, but only one of them (AGTTCAGGGAGTTGA) was conserved between humans and mice (Figure 4A). We also used 3D-footprint software to evaluate this predicted VDRE in order to look for the VDR-VDRE interaction. The analysis revealed that VDR and its heterodimeric partner, RXR-α, could recognize and bind to this conserved VDRE (Figure 4B). The study results indicate that VDR has a DNA-binding domain with an EKRR residue chain that recognizes the VDRE. On the other hand, RXR-α has two other DNA-binding domains for VDRE with EKKR and MEKKRR residues.

### 2.5. Regulation of Kv7 Expression by Vdr

To prove whether *Vdr* is involved in the regulation of kv7 channel expression, protein expression was analyzed by Western blot. Kv7.1 and Kv7.4 expression were similar in control and *Vdr* knockout mice (Figure 4C,D). However, ancillary subunit KCNE4 expression was increased in *Vdr^−^*^/*−*^ mice (Figure 4E), demonstrating that this subunit is regulated by *Vdr,* as anticipated by the bioinformatic analysis.

## 3. Discussion

In this study, we analyzed the role of *Vdr* in the pulmonary vasculature, focusing on the regulation of Kv7 channels. We found an upregulation of ancillary subunit KCNE4 in the lungs of *Vdr^−^*^/*−*^ mice. This is consistent with the finding of a consensus VDRE (a DNA-binding motif for VDR/*Vdr* and its heterodimeric partner, RXR) in the human and mouse *KCNE4* gene. Importantly, PASMCs from *Vdr^−^*^/*−*^ mice showed greater Kv7 currents and increased relaxant response to the Kv7 channel activator retigabine. Mice lacking *Vdr* did not exhibit an apparent PAH phenotype.

Vitamin D deficiency is highly prevalent in PAH patients (95%) [21,22], and low vitamin D levels are associated with poor prognosis. Moreover, *VDR* mRNA and VDR protein are also downregulated in the lungs of PAH patients [29]. In a previous study, we found that vitamin D deficiency in rats under normoxic conditions caused mild endothelial dysfunction and a moderate increase in muscularization of pulmonary arteries [23]. However, it had no effect on pulmonary pressure or right ventricular weight, indicating that, by itself, it does not trigger PH. Hence, we analyzed PAH hallmarks in WT, *Vdr^+^*^/*−*^, and *Vdr^−^*^/*−*^ mice, i.e., right ventricular hypertrophy, PA remodeling, response to vasoconstrictors, and endothelial dysfunction of the pulmonary arteries. Unfortunately, when exposed to anesthesia, mechanical ventilation, and open-chest surgery, we were unable to keep the mice alive to achieve a reliable hemodynamic recording. No changes in cardiac hypertrophy or PA function were found among the three genotypes. Furthermore, in contrast to previous findings in vitamin-D-deficient rats [23], endothelial-dependent relaxation in response to acetylcholine and vessel structure was not altered in *Vdr^−^*^/*−*^ mice. Endothelium-independent relaxation in response to sodium nitroprusside was also unchanged in *Vdr* knockout mice. Therefore, *Vdr* deletion in mice did not induce endothelial dysfunction. The absent phenotype in *Vdr^−^*^/*−*^ mice compared to the mild phenotype in vitamin-D-deficient rats may be related to the lower sensitivity of mice to pulmonary hypertensive stimuli [33]. In addition, because Kv7 channels contribute to endothelium- and NO-induced pulmonary vasodilation [34], the enhanced Kv7 currents might compensate for an otherwise reduced endothelial function. In the rat model of PAH induced by either hypoxia and the VEGF inhibitor Su5416 [23] or by monocrotaline [35], a vitamin-D-free diet aggravated the increase in mPAP and several other markers of PH. We decided not to expose the Vdr^−/−^ animals to pulmonary hypertension, i.e., hypoxia/sugen5416, as we initially aimed because it might have led to excessive weight loss and increased mortality.

A key feature of PAH is the ionic remodeling produced in the pulmonary vasculature [36,37]. The decrease in the expression of Kv1.5 and TASK-1 channels encoded by the *KCNA5* and *KCNK3* genes in PASMCs is the most remarkable in rats and humans. However, KCNK3 does not constitute a functional TASK-1 like channel in mouse PASMCs [38]. In rats, vitamin D deficiency alone reduced the currents carried by TASK-1 channels and exacerbated the decrease in both Kv1.5 and TASK-1 currents induced by hypoxia-Su5416 [23]. Surprisingly, we found that *Vdr^−^*^/*−*^ mice showed increased total K^+^ currents in PASMCs.

In PASMCs, the decrease in total K^+^ currents associated with PAH is accompanied by a seemingly compensatory increased role of Kv7 channels [14]. Thus, the Kv7 currents are increased by hypertensive stimuli following the inhibition of TASK-1 and Kv1.5 channels. Moreover, the vasodilator response to Kv7 activators is also increased, and the KCNE4 regulatory subunit is upregulated in both PAH animal models and patients. Therefore, we analyzed whether the increased total Kv current in *Vdr^−^*^/*−*^ mice might be related to increased activity of Kv7 channels. We observed that the relaxation in response to the Kv7 channel activator retigabine was significantly enhanced in *Vdr^−^*^/*−*^ mice compared to controls. This increase in relaxation in response to retigabine was not observed in heterozygous mice and was therefore uniquely dependent on total *Vdr* ablation. However, Kv7.1 channels are not sensitive to retigabine [10], which suggests that Kv7.1 might not contribute to the increased Kv7 currents in PASMCs. Furthermore, although in both control and *Vdr^−^*^/*−*^ PASMCs, the Kv7 inhibitor XE991 reduced total currents, Kv7 channel currents were higher in *Vdr* knockout animals. The contribution of Kv7 channels to total K^+^ currents was higher in the *Vdr^−^*^/*−*^ mice, but we cannot disregard the fact that other currents are also increased in these animals. These findings reveal that *Vdr* ablation produces an ionic remodeling of K^+^ channels, with an increase in Kv7 comparable to that observed in animal models and human PAH.

A previous study noted possible VDREs in the promoter of the genes coding for channels Kv7.1, Kv7.3, and KCNE4 [39]. The VDRE in the *KCNEA* gene was recognized by 3D-footprint bioinformatic analysis as a possible DNA fragment for VDR binding among other high-affinity proteins. In addition, we confirmed that *Vdr* knockout mice had increased KCNE4 expression without changes in the expression of α subunits Kv7.1 or Kv7.4. Interestingly, Jepps et al. [16] described a colocalization between KCNE4 and Kv7.4 channels and Kv7.4/Kv7.5 heteromeric channels in smooth muscle cells isolated from mesenteric arteries. In addition, the knockdown of this regulatory subunit reduced Kv7 currents. We also showed that KCNE4 increased the trafficking of Kv7.4 from cytosol to the membrane and increased its activity in PAH [14]. These data collectively show a negative regulation of KCNE4 by VDR in the pulmonary vasculature, with a negative impact on the activity of the Kv7 channels.

## 4. Materials and Methods

### 4.1. Ethical Approval

Animal experimentation was performed according to the current Spanish and European legislation relative to the use and care of laboratory animals and approved by the institutional Ethical Committee of Consejo Superior de Investigaciones Científicas (Madrid, Spain) and the regional Committee for Laboratory Animal Welfare (Comunidad de Madrid, Ref. number PROEX 396.1/21). All investigators understand the ethical principles.

### 4.2. Animal Model

*Vdr* knockout (*Vdr*^−/−^, n = 9), wild-type (WT or *Vdr*^+/+^, n = 8), and heterozygous (*Vdr*^−/+^, n = 4) male mice were obtained by crossing *Vdr*^−/+^ mice. They were originally generated by Dr. Marie Demay (Harvard Medical School, Boston, MA, USA) and kindly donated to us to breed our own colony [31,32]. Mice were genotyped (Figure 1A) as described in protocol 22517 (http://www.jax.org, accessed 28 July 2023). To normalize the blood mineral ion levels of the *Vdr*-ablated mice, the animals were fed a g-irradiated diet (TD96348, Teklad, Madison, WI, USA) containing 2% calcium, 1.25% phosphorus, and 20% lactose from 19 days of age [31,32]. All mice were kept in a virus- and parasite-free barrier facility at 22 ± 1 °C, exposed to a 12 h light and dark cycle, and allowed free access to autoclaved water and chow. At 4 months of age, mice were sacrificed by decapitation under anesthesia (80 mg/kg ketamine and 8 mg/kg xilacine i.p.). Throughout the subsequent dissection, the lungs and heart were removed and placed in cold (4 °C) Krebs–Henseleit solution (KHS) bubbled with a 95% O_2_-5% CO_2_ mixture (pH = 7.4). Resistance pulmonary arteries were dissected on the same day for vascular reactivity and patch-clamp electrophysiology, and the remaining tissues were embedded in paraformaldehyde saline solution (4%, PFA) or frozen and stored at −80 °C to perform histological and molecular analysis, respectively.

### 4.3. RV Hypertrophy and Lung Histology

Hearts were excised, and the right ventricle (RV) and the left ventricle plus septum (LV+S) were dissected and weighed separately. The Fulton index (RV/(LV+S)) was calculated to assess right ventricular hypertrophy. The left lung was inflated in situ with PFA through the left bronchus and embedded in paraffin. All sections were cut at 5 µm, stained with hematoxylin and eosin, and examined by light microscopy. Elastin was visualized by its green autofluorescence. For quantification of pulmonary vascular remodeling, PA (25–250 µm outer diameter; OD) was analyzed in a blinded fashion and categorized as muscular, partially muscular, or non-muscular as previously described [40,41,42]. Muscular or partially muscular arteries were defined as those that had a complete or interrupted muscle coat lying between an internal and external elastic lamina. Non-muscular arteries were defined as vessels with no muscle in their wall [42]. The medial wall thickness was calculated using ImageJ software as the difference between the external elastic lamina diameter and the internal lamina diameter.

### 4.4. Arterial Reactivity

For vascular function, PA rings (1.7–2 mm long, ~0.8 mm internal diameter) were mounted in a wire myograph with KHS, maintained at 37 °C, and bubbled with a mixture of 21% O_2_ and 5% CO_2_. Vessels were stretched to achieve an equivalent transmural pressure of 30 mmHg. After equilibration, arterial rings were first stimulated with KCl (80 mmol/L). Then, endothelial function in PA rings precontracted with phenylephrine (1 µmol/L) was assessed by the cumulative addition of acetylcholine (Ach, 1 nmol/L to 10 µmol/L). After washout, the dose–response curve of serotonin (5-HT, 30 nmol/L to 30 µmol/L) was generated by cumulative drug addition, followed by the concentration–response curve of sodium nitroprusside (SNP, 10 pM to 10 µM) by cumulative drug addition. Finally, after washing, the rings were precontracted again to 10 µM serotonin (5HT) to generate a final dose–response curve of retigabine (10 nM to 30 µM).

### 4.5. Electrophysiological Studies

PASMCs were enzymatically isolated as previously described [43]. Whole-cell currents were recorded on an Axopatch 200B amplifier, filtered at 2 kHz, digitized at 5 kHz (Digidata 1322A; Axon Instruments, Burlingame, CA, USA), and stored on a computer for subsequent offline analysis with Clampfit 10.3 software (Molecular Devices, Sunnyvale, CA). Myocytes were superfused with an external Ca^2+^-free Hepes solution (see above) and a Ca^2+^-free pipette (internal) solution containing (in mmol/L) KCl 110, MgCl_2_ 1.2, Na_2_ATP 5, HEPES 10, and EGTA 10 (pH adjusted to 7.3 with KOH). Kv currents were evoked following the application of 200 ms depolarizing pulses from −60 mV to +60 mV in 10 mV increments. To characterize the contribution of Kv7 channels to the total Kv current, cells were exposed to the selective inhibitor XE991 (10 µmol/L). All experiments were performed at room temperature (22–24 °C). Freshly isolated cells were stored in ice-cold isolation medium and used the same day for up to 5 h.

### 4.6. Identification of VDREs in the KCNE4 Gene by In Silico Analysis

To identify VDREs in the *KCNE4* gene, we searched the database of consensus DR3 and ER6 elements, as well as DR3 elements containing single-nucleotide substitutions [39]. The predicted VDRE in the *KCNE4* gene promoter conserved between humans and mice was subjected to in silico analysis using 3D-footprint, a database for the structural analysis of protein–DNA complexes [23,44]. In addition to predicted proteins that might recognize a certain DNA motif, it provides a string of concatenated interface residues (also known as proteic interface signatures) and the binding specificities in the VDRE sequence.

### 4.7. Western Blot

Lungs were homogenized with a lysis buffer containing Trizma preset crystals (pH 7.5), 1 mol/L DL-dithiothreitol (DTT), and 1% NP40 and supplemented with protease (Protease inhibitor cocktail tablets (Roche Diagnosis GmbH, Vienna, Austria) and phosphatase (PhosSTOP, Roche Diagnostics GmbH) inhibitor cocktail in a Tissuelyser device (Qiagen, Hilden, Germany). After four short pulses (30 s, stopping 15 s between each pulse) of homogenization, the lysates were centrifuged for 10 min at 10,000 rpm. The protein concentration was determined by a colorimetric assay based on the Lowry method (Biorad, Hercules, CA, USA). Twenty-five µg of proteins were run on sodium dodecyl sulphate-polyacrylamide electrophoresis, and proteins were transferred to polyvinylidene difluoride membranes (Biorad, CA, USA). Membranes were blocked by one-hour incubation with 5% non-fat milk TBST (0.5 M Tris pH 7.5; 1.5 M NaCl; 0.1%Tween© 20) and incubated overnight at 4 °C with primary rabbit antibodies against Kv7.4 (Santa Cruz Biotechnology, TX, USA; Cat# sc-50417; 1:200), Kv7.1 (Alomone, Jerusalem, Israel; Cat# APC-022; 1:200), and KCNE4 (Atlas antibodies, Bromma, Sweden; Cat# HPA011420; 1:100). Membranes were subsequently incubated with the appropriate secondary antibodies conjugated with horseradish peroxidase at room temperature for one hour (Merck, Darmstadt, Germany; Cat# 401315-M, 1:10,000). Antibody binding was detected by an ECL system (Amersham Pharmacia Biotech, Amersham, UK or SuperSignal West Fento Chemiluminescent Substrate, Thermo Scientific, Waltham, MA, USA). Blots were imaged using an Odissey Fc System (Li-COR Biosciences, Lincoln NE, USA) and quantified by densitometry using Quantity One software. Results were normalized relative to vinculin (Santa Cruz Biotechnology, Dallas TX, USA; Cat# Sc-25336; 1:1000) signal intensity.

### 4.8. Statistics

Analysis was performed using GraphPad Software v8 (GraphPad Software Inc., Boston, MA, USA). All data were tested for normal distribution using the Shapiro–Wilk test, and parametric or non-parametric statistics were used as appropriate. Data are presented as means ± SEM of 4–8 animals. For this study, we used a *t*-test and one-way or two-way ANOVA analysis followed by Bonferroni post hoc test as appropriate. Differences in pulmonary artery muscularization were analyzed by a chi-square test. *p* values of less than 0.05 were considered statistically significant.

## 5. Conclusions

In conclusion, we observed that the absence of *Vdr* alone did not induce a PAH phenotype. However, it enhanced the Kv7 channel activity and downregulated KCNE4 in the pulmonary vasculature, which resembles the ionic remodeling observed in PAH patients.

## Data Availability

Data are contained within the article, and raw data are available upon request.

## Supplementary Materials

The following supporting information can be downloaded at: https://www.mdpi.com/article/10.3390/ijms241512350/s1.
