# Supplementary material for: Vitamin D Receptor Deficiency Upregulates Pulmonary Artery Kv7 Channel Activity
Source: Int J Mol Sci. 2023 Aug 2;24(15):12350. doi: 10.3390/ijms241512350 (PMC10418734; doi:10.3390/ijms241512350)
Supplement: Supplementary file 1 [file ijms-24-12350-s001.zip › ijms-2450125-supplementary.pdf]

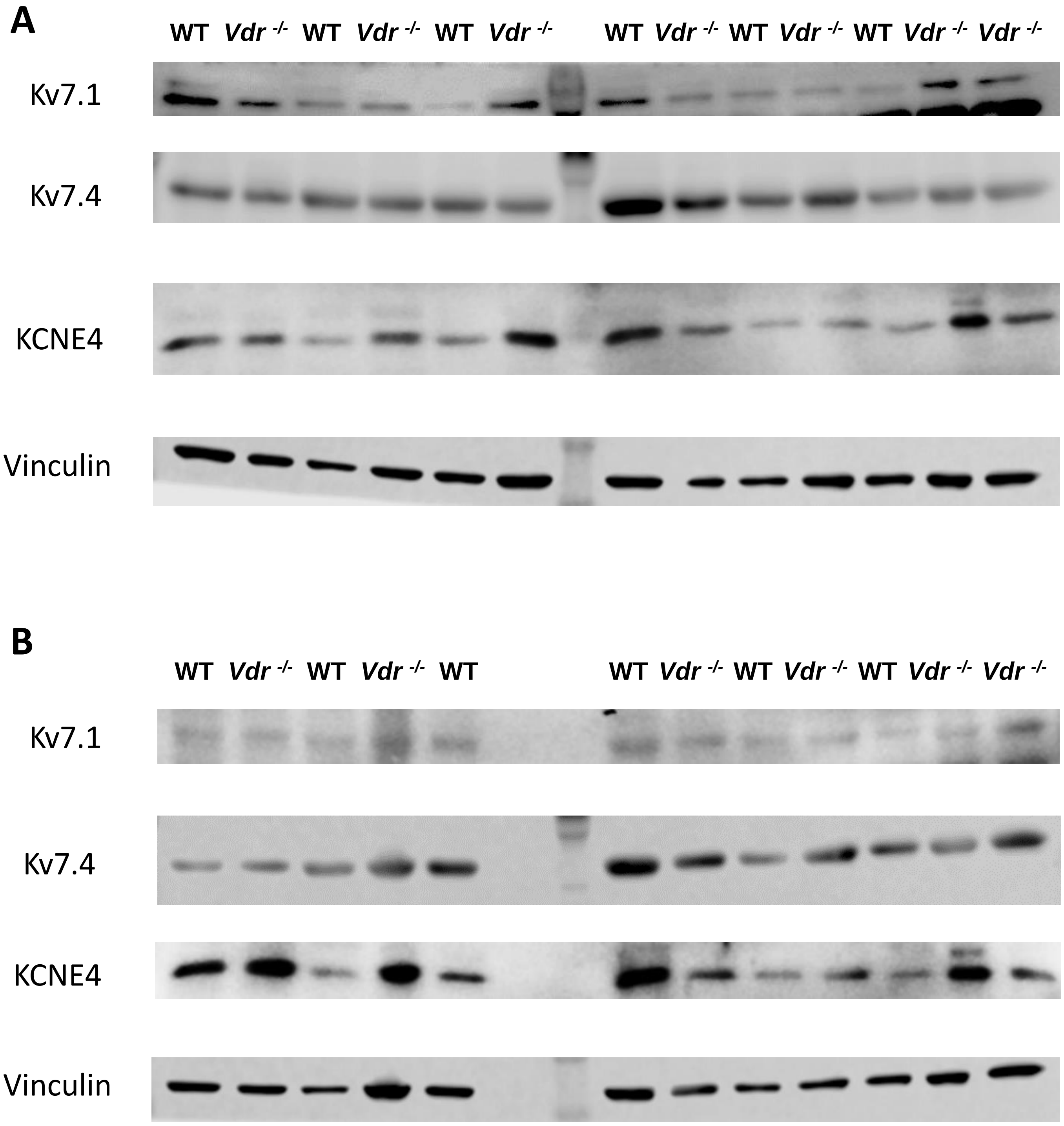

Figure S1. Protein expression of Kv7.1, Kv7.4 and KCNE4 in the lungs from wild-type and *Vdr* knockout mice. A and B) Original Western blots in duplicate. The averaged densitometric values normalized to vinculin expression are shown in Figure 4. Note that proteins were analysed in the same membranes and, therefore, share the vinculin references.
